# MIB-ANet: A novel multi-scale deep network for nasal endoscopy-based adenoid hypertrophy grading

**DOI:** 10.3389/fmed.2023.1142261

**Published:** 2023-04-14

**Authors:** Mingmin Bi, Siting Zheng, Xuechen Li, Haiyan Liu, Xiaoshan Feng, Yunping Fan, Linlin Shen

**Affiliations:** ^1^Department of Otolaryngology, The Seventh Affiliated Hospital of Sun Yat-sen University, Shenzhen, China; ^2^College of Computer Science and Software Engineering, Shenzhen University, Shenzhen, China; ^3^AI Research Center for Medical Image Analysis and Diagnosis, Shenzhen University, Shenzhen, China

**Keywords:** adenoid hypertrophy, nasal endoscopy, deep learning, medical image classification, convolutional neural networks

## Abstract

**Introduction:**

To develop a novel deep learning model to automatically grade adenoid hypertrophy, based on nasal endoscopy, and asses its performance with that of E.N.T. clinicians.

**Methods:**

A total of 3,179 nasoendoscopic images, including 4-grade adenoid hypertrophy (Parikh grading standard, 2006), were collected to develop and test deep neural networks. MIB-ANet, a novel multi-scale grading network, was created for adenoid hypertrophy grading. A comparison between MIB-ANet and E.N.T. clinicians was conducted.

**Results:**

In the SYSU-SZU-EA Dataset, the MIB-ANet achieved 0.76251 F1 score and 0.76807 accuracy, and showed the best classification performance among all of the networks. The visualized heatmaps show that MIB-ANet can detect whether adenoid contact with adjacent tissues, which was interpretable for clinical decision. MIB-ANet achieved at least 6.38% higher F1 score and 4.31% higher accuracy than the junior E.N.T. clinician, with much higher (80× faster) diagnosing speed.

**Discussion:**

The novel multi-scale grading network MIB-ANet, designed for adenoid hypertrophy, achieved better classification performance than four classical CNNs and the junior E.N.T. clinician. Nonetheless, further studies are required to improve the accuracy of MIB-ANet.

## 1. Introduction

Adenoid hypertrophy is a common disease in children with otolaryngology diseases. A meta-analysis showed that the prevalence of adenoid hypertrophy in children and adolescents was 34.46% (1). Adenectomy or adenotomy is the first-recommended therapy for sleep disordered breathing in children, with “adenoid faces” (2) and other growth and development problems. Clinically, surgical indication is on the basis of the grading of adenoid hypertrophy. There are four main grading standard of adenoid hypertrophy based on nasal endoscopy, i.e., Clemens grading standard (3), Cassano grading standard (4), Parikh grading standard (5), and ACE grading system (6). Among which, Parikh grading standard, which was reported on Otolaryngol Head Neck Surg. in 2006, grades adenoid hypertrophy by evaluating the adjacent structure of adenoid tissue contact, which can reflect the degree of blockage in the Eustachian tube and can be related to the meaning of the surgery. However, long-time reading of different images is a tedious work and may cause misdiagnosis, especially for interns without experiences. Creating an artificial intelligence deep network for nasal endoscopy-based adenoid hypertrophy grading is meaningful.

In recent years, many deep learning methods, especially convolutional neural networks (CNNs), have been applied in the medical image domain (7–12). For adenoid hypertrophy, Shen et al. (13) collected 688 lateral cranial X-ray images of patients with adenoid hypertrophy, and divided these images into training set (488), validation set (64) and test set (116). This deep learning model calculated the AN ratio (AN ratio, where A is the absolute size of the adenoid and N is the size of the nasopharyngeal space) to grade adenoid hypertrophy. Liu et al. (14) collected 1,023 lateral cranial X-ray images, and proposed a deep learning model based on VGG16 to grade adenoid hypertrophy. In the clinic, nasoendoscope is a simple, economical, readily available, and reproducible way to diagnose adenoid hypertrophy. Compared to lateral cranial X-ray, nasoendoscope requires no radiation and provides good view to investigate the distance relationship between adenoid and adjacent structures. However, to the best of our knowledge, there is no deep learning research available to help grade endoscopic images of adenoid hypertrophy.

Inspired by the success of previous works in detection and classification of medical endoscopic images, in this study, we assumed that the adenoid hypertrophy grading could also benefit from deep learning techniques. Toward this end, we acquired a large collection of nasal endoscopic images to build a novel MIB-ANet model and assessed its performance.

## 2. Materials and methods

### 2.1. SYSU-SZU-EA dataset

We reviewed the nasoendoscopic images of patients who underwent routine clinical screening for nasal diseases at the Seventh Affiliated Hospital of Sun Yat-sen University (Shenzhen, China), between December 2019 and May 2021. All of the images in SYSU-SZU-EA Dataset were original nasoendoscopic images, without artificial light, zoom, and optical amplification restrictions. We only choose images capturing adenoid residue or adenoid hypertrophy. There was no limitation for age, gender, or whether to combine chronic rhinosinusitis or other diseases. This dataset consists of 3,179 images. All images were captured using a rigid 0-degree 2.7 mm nasoendoscope and endoscopic capture recorder (Wolf, Tuttlingen, Germany), equipped with high-performance medical imaging workstation. All of the images were saved with JPG format consisting of red, green, and blue color channels and had widths and heights ranging from 700 and 1,000 pixels. All the patients had signed informed consent before nasoendoscopy.

### 2.2. Grading method of adenoid hypertrophy

**Figure 1:**
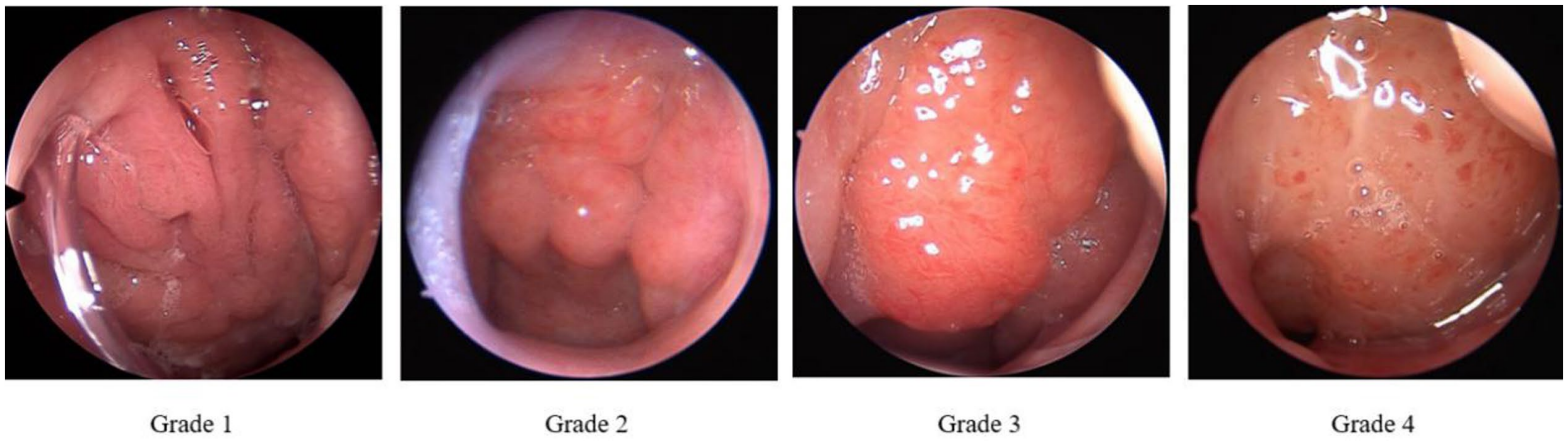
Examples of 4 grades adenoid nasoendoscopic images according to Parikh grading standard in the SYSU-SZU-EA dataset.

There are four main grading standard of adenoid hypertrophy, i.e., Clemens grading standard (3), Cassano grading standard (4), Parikh grading standard (5), and ACE grading system (6). Among which, Parikh grading standard grades adenoid hypertrophy by evaluating the adjacent structure of adenoid tissue contact, which can reflect 3D structure and requires few parameters, and is convenient for clinical evaluation and deep learning. Therefore, in this work Parikh grading standard were chosen as the grading method. Table 1 shows the grading method of adenoid hypertrophy and the detailed numbers of images of four grades. Adenoid hypertrophy is divided into 1–4 grades according to whether the adenoid tissue contacted or pressed the Eustachian tube pillow, vomer bone, and soft palate in a relaxed state. Figure 1 shows four example adenoid images with grades 1 to 4 in the SYSU-SZU-EA Dataset. Three E.N.T. clinicians, including one senior E.N.T. clinician, one intermediate E.N.T. clinician and one junior E.N.T. clinician were employed for data annotation.

**Table 1:** Details of Parikh grading standard and data distribution of training set, validation set, and test set in SYSU-SZU-EA dataset.

Grade	Adjacent structure of adenoid tissue contact	Training set	Validation set	Test set	Number
1	None	428	122	228	778
2	Torus tubarius	576	158	276	1,010
3	Torus tubarius, vomer	492	104	355	951
4	Torus tubarius, vomer, palate (at rest)	250	53	137	440
Total		1746	437	996	3,179

### 2.3. Preprocessing

*Computer implementation environment*: The neural network models were coded in Python (version 3.7.6, 64 bit) using the open-source Pytorch (version 1.8.1) library and tested on Intel (R) Xeon (R) Gold 6,132 CPU @2.60GHz and a Tesla V100. Due to limited GPU resources, all images were resized to 256 × 256 pixels. In the training phase, we used a learning rate of 0.0001 and a batch size of 32 in the Adam optimizer, and used the “StepLR” with step size of 10, gamma of 0.9 to decay the learning rate. In addition, we employed random vertical flip, random horizontal flip, and random rotation on the input images to augment the dataset in training.

*Data distribution of training set, validation set and test set*: We randomly divided the 2,183 adenoid images into training set and validation set. The ratio of the image number of training set to the validation set is 4:1. In order to ensure that the number of adenoid images at each grade in the training set is sufficient, the dividing ratio for grade 1 and 2 was set as approximately 4:1, and the dividing ratio for grade 3 and 4 was set as approximately 5:1. For testing set, 996 images were graded by 3 E.N.T. clinicians with different experiences and the final result was determined based on majority voting. The detailed distribution of adenoid hypertrophy images with different grades in training set, validation set, and test set is shown in Table 1.

### 2.4. The novel multi-scale grading network: MIB-ANet

**Figure 2:**
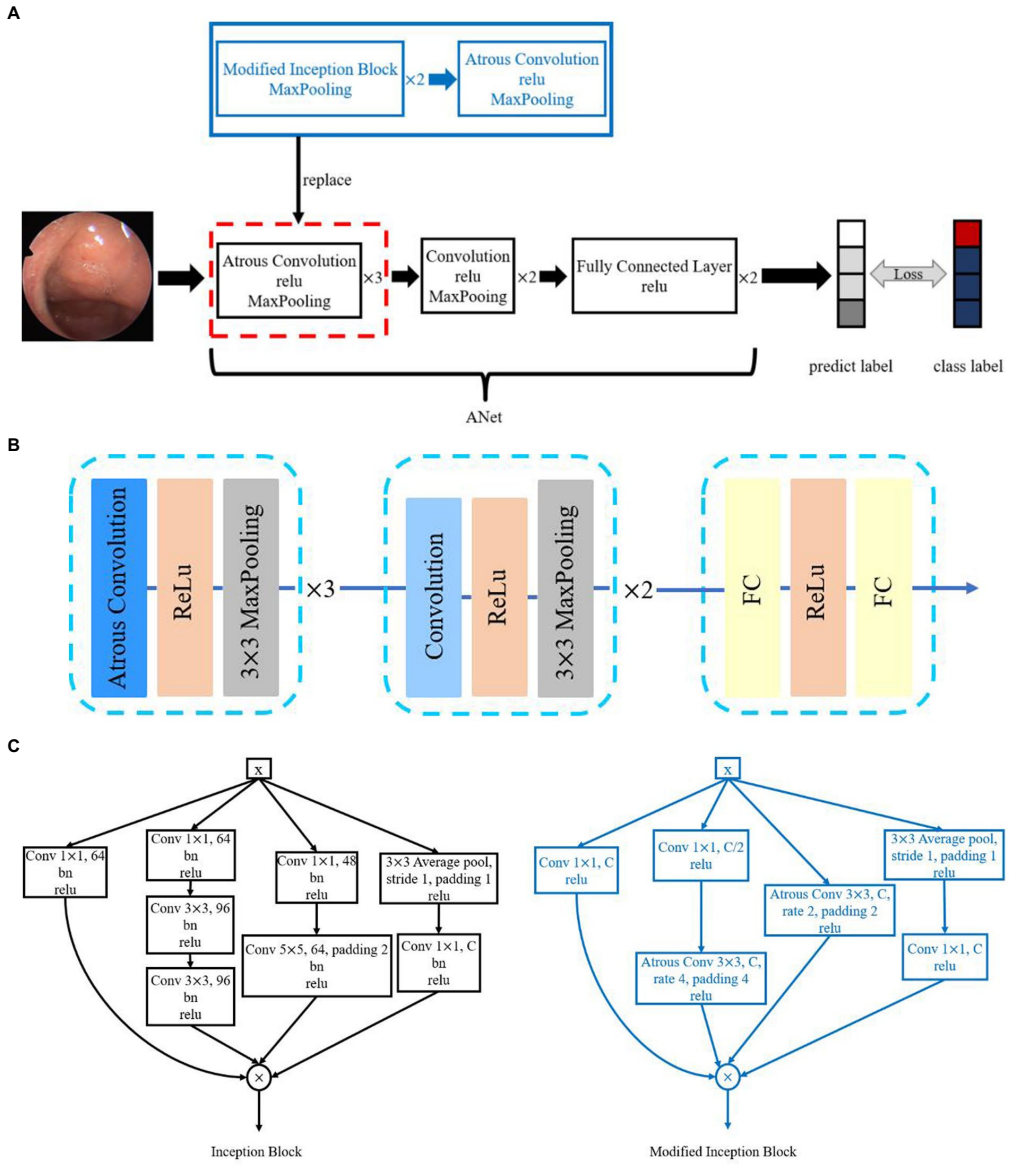
**(A)** The overview of the proposed MIB-ANet architecture. **(B)** The architecture of ANet. **(C)** The architecture of Inception Block and Modified Inception Block.

In this paper, we designed a framework, MIB-ANet, for adenoid hypertrophy classification. As shown in Figure 2A, the proposed MIB-ANet consisted of two modules, the backbone network—ANet and Modified Inception Block (MIB). MIBs and ANet were integrated as MIB-ANet by replacing the first two layers of ANet (red dotted box) with MIBs (blue box), whose details are shown in Figures 2B,C and Supplementary B.

### 2.5. Performance evaluation

In this study, four classic CNNs, i.e., AlexNet (15), VGG16 (16), ResNet50 (17), and GoogleNet (18), were employed for performance comparison. Details of the structure of four classic CNNs and ANet are described in Supplementary A. Accuracy, F1 score and confusion matrix were adopted as the evaluation metrics of classification performance. Definition of Evaluation Metrics are described in Supplementary B. Details of ablation study for Classification Performance evaluation are described in Supplementary C. We also used the Class Activation Map (CAM) (19) to visualize the attention map of different CNNs, which can highlight the regions of interest of different models. The comparison of the performance of MIB-ANet, ANet and four classic CNNs are showed in Supplementary D.

### 2.6. Comparison between MIB-ANet and E.N.T. clinicians

We compared the diagnostic performance of MIB-ANet with three E.N.T. clinicians. While the senior E.N.T. clinician has more than 20 years of experience in nasal endoscopy, the intermediate and junior E.N.T. clinician has approximately 8 years and 5 years of experience in nasal endoscopy, respectively. They conducted blind assessments of 996 images in testing set and the final result was determined based on majority voting. We compared MIB-ANet with human experts using F1 score and accuracy.

### 2.7. Ethics

The study was approved by the ethical review board of the Seventh affiliated Hospital of Sun Yat-sen University (no. KY-2022-008-01).

### 2.8. Statistical analysis

ROC curves were adopted as the evaluation metrics of classification performance, which were coded in Python (version 3.7.6, 64 bit). Wilcoxon signed-rank test was used to analyze the difference between two paired samples of ordinal categorical variables, which was performed by SPSS 17.0. All tests were two-sided, and *p* < 0.05 was considered as statistically significant.

## 3. Results

### 3.1. Comparison based on F1 score and accuracy

We compared the performance of MIB-ANet to E.N.T. clinicians. Since the test set was annotated by 3 E.N.T. clinicians independently, the ground truth was determined based on majority voting and a face-to-face discussion of these 3 E.N.T. clinicians. Therefore, we evaluated the performance of each doctor by calculating the F1 score and accuracy of their diagnostic results with the voted ground truth. Table 2 shows the performance of MIB-ANet and 3 E.N.T. clinicians. From Table 2, we can see that MIB-ANet achieved at least 6% higher F1 score and 4% higher accuracy than the junior clinician, and achieved much higher diagnosing speed than human experts, e.g., at least 80 times faster than the senior clinician. Table 2 also shows the detailed *Z* and *p* value between the voted ground truth and MIB-ANet or 3 E.N.T. clinicians. Since the classification results were ordinal categorical variables, two-sided Wilcoxon signed-rank test was employed to analyze the difference between two paired samples. As we know, *p* value indicates the statistical significance and *Z* value indicates the tendentiousness. The *p* value of MIB-ANet was 0.188, which showed that there was no significant statistical difference between the voted ground truth and MIB-ANet. However, the *p* values of 3 E.N.T. clinicians were smaller than 0.05, which meant that there were significant statistical differences between the voted ground truth and 3 E.N.T. clinicians. The *Z* values of both MIB-ANet and 3 E.N.T. clinicians were smaller than zero, which meant that both clinicians and deep model tended to make prediction of higher grade. Compared to 3 clinicians, MIB-ANet achieved the smallest absolute *Z* value, which meant that the prediction of MIB-ANet was more objective.

**Table 2:** Performance of MIB-ANet to E.N.T. clinicians.

	Evaluation metrics		Time (s)	vs. Ground truth	
	F1 score	Accuracy		*Z*	*p* value
Senior clinician	**0.89013**	**0.89558**	4 ~ 8	−6.962	0.000*
Intermediate clinician	0.80555	0.80422	5 ~ 11	−8.307	0.000*
Junior clinician	0.69867	0.72490	7 ~ 13	−5.618	0.000*
MIB-ANet	0.76251	0.76807	**0.05**	−1.316	0.188

We used“bold”to highlight the best performance of the variable.

### 3.2. Comparison based on ROC curve and confusion matrices

**Figure 3:**
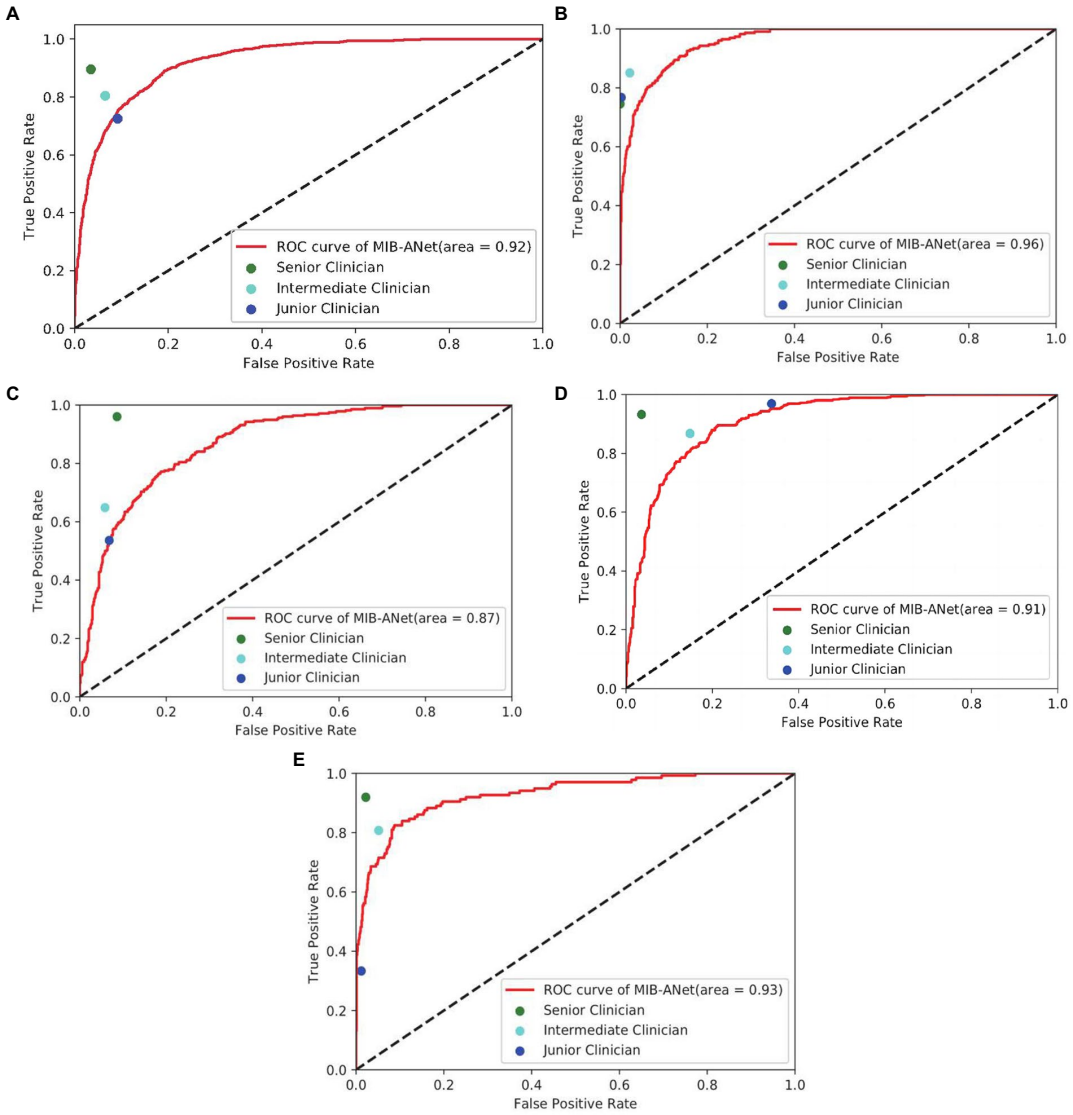
**(A–E)** show the overall micro-average ROC curve of MIB-ANet and that for grade 1 to 4 adenoid hypertrophy respectively, compared with TPR and FPR of 3 E.N.T. clinicians.

Figure 3 shows the micro-average ROC curve of MIB-ANet and different grade. True Positive Rate (TPR) as well as False Positive Rate (FPR) of 3 E.N.T. clinicians. For points in ROC curve, the closer to the upper left corner, the better grading performance. From Figure 3A, we can see that the senior clinician (green point) showed the best grading performance. MIB-ANet (red curve) showed performance between intermediate clinician (aqua point) and junior clinician (blue point). From Figures 3B,D, we can see that for grade 1 and grade 3 adenoid images, 3 E.N.T. clinicians showed better performance than MIB-ANet (All of points are located above the curve of MIB-ANet). From Figures 3C,E, we can see that for grade 2 and grade 4 adenoid images, MIB-ANet showed better performance than junior clinician (blue point is located below the red curve), while showed worse performance than senior clinician and intermediate clinician (green point and aqua point are located above the red curve).

**Figure 4:**
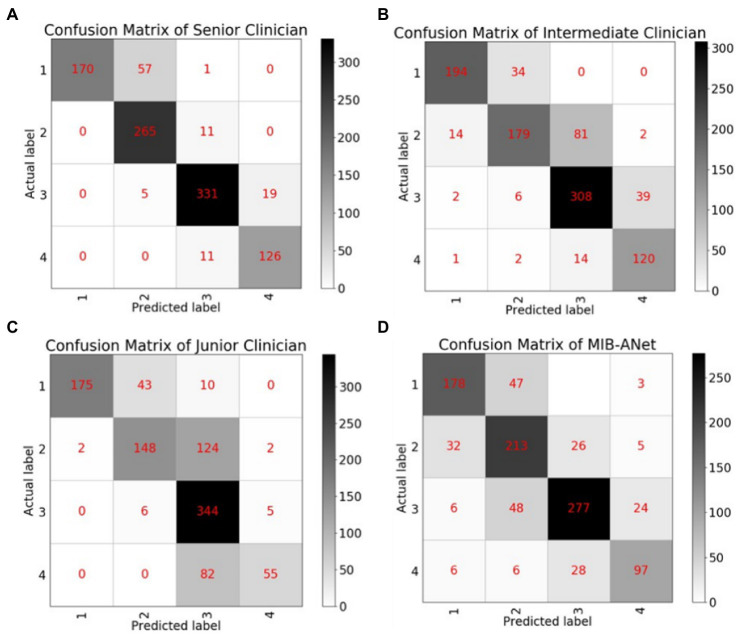
The confusion matrices of MIB-ANet and 3 clinicians. **(A–D)** Show the confusion matrix of senior clinician, intermediate clinician, junior clinician, and MIB-ANet, respectively.

Figure 4 shows the confusion matrices of MIB-ANet and human experts. From these matrices, we can calculate that for grade 1 adenoid images, the accuracy of 3 E.N.T. clinicians were roughly the same and higher than that of MIB-ANet. For grades 2, 3, and 4 adenoid images, senior clinician achieved the best accuracy, which were 0.92671, 0.95281, and 0.96988, respectively. MIB-ANet achieved better accuracy (0.86747) than intermediate clinician (0.85743) for grade 3 adenoid images. And for grades 2, 3, and 4 adenoid images, MIB-ANet achieved better accuracy (0.83534/0.86747/0.92771) than junior clinician (0.82229/0.77209/0.91064).

### 3.3. Comparison based on heatmap visualization

**Figure 5:**
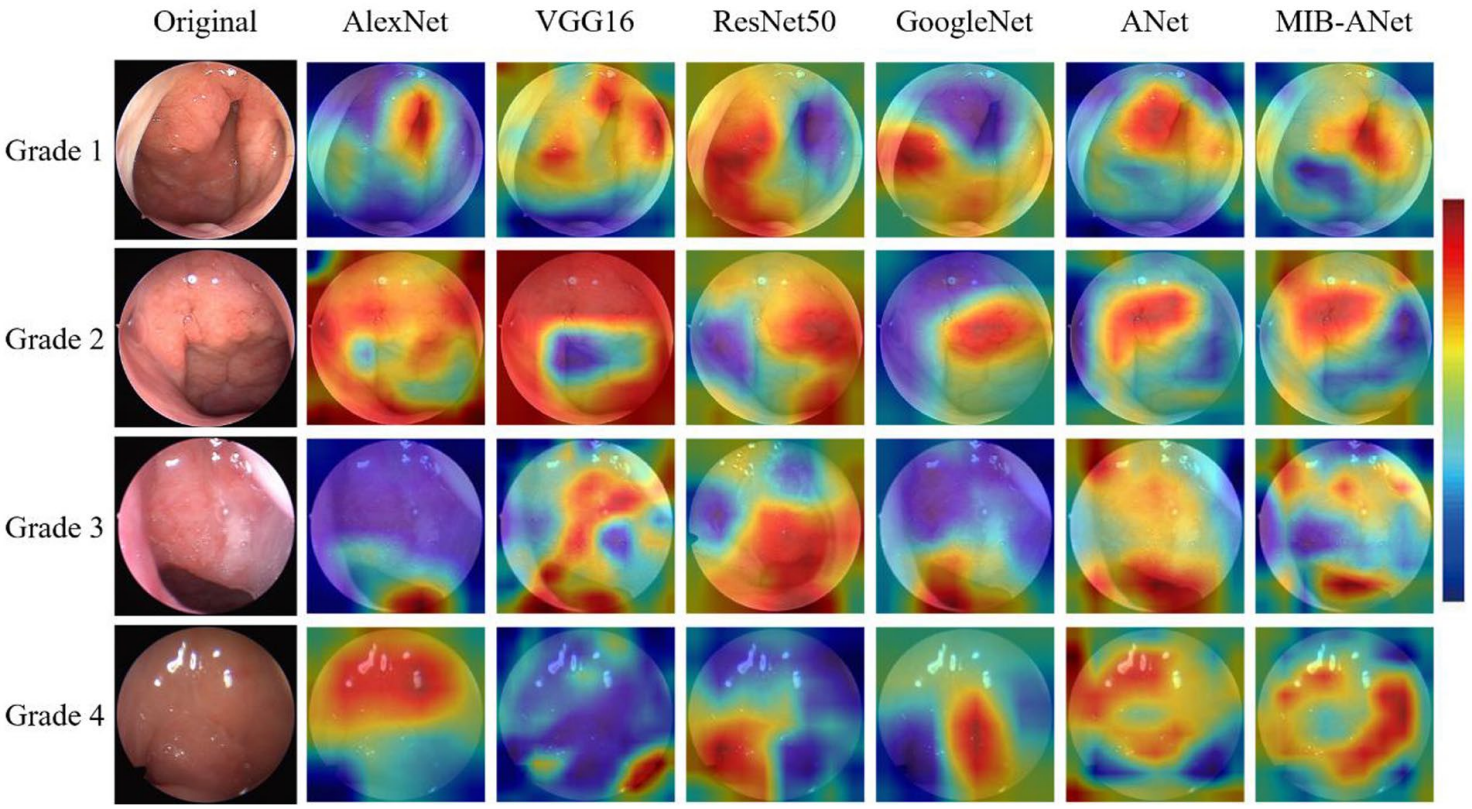
The heatmaps of different deep networks for adenoid hypertrophy prediction. The first column shows the original adenoid images. The second, third, fourth, fifth, sixth, and seventh columns show the heatmaps of AlexNet, VGG16, ResNet50, GoogleNet, ANet and MIB-ANet, respectively.

Figure 5 shows the heatmaps overlaid on adenoid nasoendoscopic images, which denotes attention map of different neural networks according to weighting of all pixels dictated by CAM. From Figure 5, we can see that, for grade 1 and grade 2, AlexNet, VGG16, ANet, and MIB-ANet tended to focus on whether the adenoid tissue is in contact with torus tubarius; ResNet50 and GoogleNet tended to focus on the adenoid area and whether adenoids were in contact with vomer. For grade 3 and grade 4, VGG16 and ResNet50 tended to focus on whether adenoids were in contact with soft palate. For grade 3, AlexNet, GoogleNet, and ANet tended to focus on the size of the airway (to some extent, the larger the adenoid, the smaller the airway space). For grade 4, AlexNet, GoogleNet, and ANet tended to focus on the adenoid area. In contrast, MIB-ANet can always focus on whether adenoids were in contact with adjacent tissues, which meant that the prediction made by MIB-ANet was based on the contact between adenoids and adjacent tissues, which was the same as how E.N.T. clinician make a decision14. The heatmaps intuitively explain why MIB-ANet has the best performance among all networks.

### 3.4. Performance of different grades

**Figure 6:**
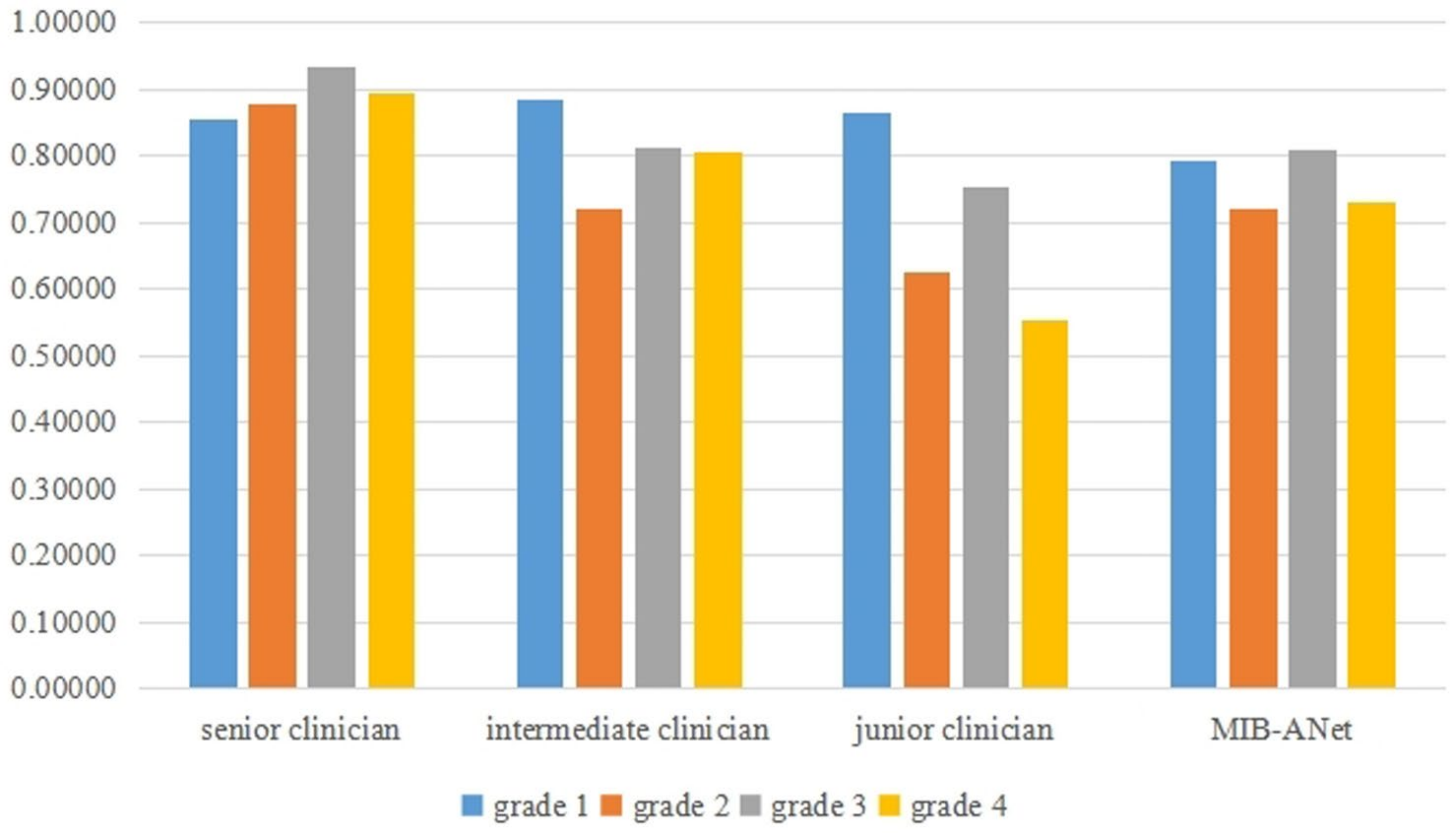
The F1 score of different grades for MIB-ANet, senior clinician, intermediate clinician, and junior clinician.

Figure 6 shows the F1 score of different grades for MIB-ANet, senior clinician, intermediate clinician, and junior clinician. From Figure 6, we can see that senior clinician showed the best classification performance among 3 E.N.T. clinicians. For grades 2, 3, and 4 adenoid images, senior clinician achieved 10–30% higher F1 score than intermediate clinician and junior clinician, and for grade 1 adenoid images, senior clinician showed comparable F1 score to intermediate clinician and junior clinician. Compared to 3 E.N.T. clinicians, MIB-ANet achieved comparable F1 score to intermediate clinician for grade 2 and 3 adenoid images, which was 9 and 5% higher than junior clinician, respectively. For grade 4 adenoid images, MIB-ANet achieved 7% lower F1 score than intermediate clinician, but 17% higher than junior clinician. For grade 1 adenoid images, MIB-ANet achieved lower F1 score than 3 E.N.T. clinicians, but only 5% lower than senior clinician. Overall, the performance of MIB-ANet was better than junior clinician and close to intermediate clinician.

## 4. Discussion

Clinically, the grading of adenoid hypertrophy is important for surgical indication. There are several medical examinations to evaluate adenoid hypertrophy, such as lateral cranial X-ray, nasoendoscopy, cone-beam computed tomography (CBCT) (20), MRI, and 3D printed model (21). Nasal endoscopy is a radiation-free, safe, and convenient operation, which is routinely used for adenoid hypertrophy grading examination. In this work, we built SYSU-SZU-EA nasoendoscopic image dataset and proposed a novel efficient deep neural network, MIB-ANet, for adenoid hypertrophy classification. To the best of our knowledge, this is the first deep learning research to address the grading of endoscopic images of adenoid hypertrophy. The experimental results showed that our network achieved better classification performance than four classical CNNs, i.e., AlexNet, VGG16, ResNet50, and GoogleNet. When compared to three E.N.T. clinicians, MIB-ANet achieved much higher (80× faster) diagnosing speed, with a grading performance better than the junior E.N.T. clinician.

In recent years, many deep learning methods, especially convolutional neural networks (CNNs), have been applied in the medical image domain. Girdler et al. (22) categorized 297 nasoendoscopic images by using the CNN model of ResNet-152 for automated detection and classification of nasal polyps and inverted papillomas. Overall accuracy of 0.742 ± 0.058 was achieved. Yang et al. (23) developed a cascaded under-sampling ensemble learning method (CUEL) to prevent and diagnose clinical rhinitis, which achieved 90.71% average accuracy on 2,231 clinical rhinitis instances. The current deep learning network is mostly used for the diagnosis of diseases. Even in the field of capsule endoscopic images with a large number of deep learning researches, little work is conducted to classify the degree of disease. In this study, we focused on the clinical requirement of adenoid hypertrophy grading, rather than disease diagnosis. At the same time, more detailed assessment, such as the nasal mucosal inflammation state, the size degree of polyps, and grading of adenoid hypertrophy, can lead to the creation of an automatic nasal endoscopy reporting system, which can reduce the burden of E.N.T clinicians and improve efficiency and accuracy of reading caused by visual fatigue.

Usually, different network models are suitable for different data sets, and the design of network structure should be based on the characteristics of data sets. Medical data sets are different from data sets collected in daily life, such as ImageNet, and contain much smaller number of images. However, the classical deep learning model has a large number of parameters, which is easy to over fit when these models are trained using small data sets in the medical field. Therefore, in order to avoid the over fitting problem in the classification of adenoid hypertrophy, we tried to reduce the amount of model parameters when designing the network structure. In addition, compared with natural images, nasoendoscopic images are characterized by more concentrated color distribution (overall red color), more abundant texture features (tissue blood vessels, dense tissue distribution), and large differences in size and shape among different types of adenoid. The classical deep learning model cannot well extract both low-level and high-level adenoid hypertrophy features. In order to solve this problem, we proposed ANet to extract high-level adenoid hypertrophy features using dilated convolutions. Based on ANet, we proposed MIB-ANet with convolution kernels of different sizes to extract both low-level and high-level adenoid hypertrophy features. The performance of ANet and MIB-ANet was better than four classic CNNs. In addition, the experimental results showed that MIB-ANet can achieve a grading performance better than the junior E.N.T. clinician with much higher diagnosing speed.

However, some limitations in our study should be mentioned. Firstly, we annotate the ground truth label of testing set according to the evaluation results of 3 E.N.T. experts with the principle of majority voting, which might still generate some incorrect labels. Further manual data cleaning and more reasonable annotation process, e.g., intraoperative evaluation of adenoid size, are required (24). Secondly, when MIB-ANet was used to grade adenoid hypertrophy, the model tended to fit the size of adenoid. When the image of adenoid collected by endoscopic technician is not standard (for example, the endoscope is close to the adenoid when collecting the adenoid image), MIB-ANet is easy to predict a higher grade. Therefore, the E.N.T clinicians are suggested to draw the boundary of the designated anatomical structure or attention area by using some software like imageScope, which can further improve the performance. At the same time, enlarging the database and building up a multicenter data platform are also helpful to improve the model. Finally, in this study, we only focused on adenoid hypertrophy grading on nasoendoscopic images. In the future, we can further add labels of other nasopharyngeal diseases, such as nasopharyngeal carcinoma and nasopharyngitis, and develop a comprehensive classification model for nasal disease diagnosis.

## Data availability statement

The raw data supporting the conclusions of this article will be made available by the authors, without undue reservation.

## Ethics statement

The studies involving human participants were reviewed and approved by Ethical Review Board of the Seventh affiliated Hospital of Sun Yat-sen University. Written informed consent to participate in this study was provided by the participants’ legal guardian/next of kin. Written informed consent was obtained from the minor(s)' legal guardian/next of kin for the publication of any potentially identifiable images or data included in this article.

## Author contributions

MB was involved in conception, design, data collection, data analysis, and interpretation, and drafted manuscripts. SZ was involved in conception, network design, data analysis, and interpretation, and drafted manuscripts. YF, XL, and LS was involved in conception and design, and made critical revisions to the manuscript. HL and XF participated in data collection and adenoid hypertrophy grading. All authors have given final recognition and agreed to be responsible for all aspects of the work.

## Funding

This work was supported by Sanming Project of Medicine in Shenzhen under Grant No. SZSM202111005; Shenzhen Fundamental Research Program (Grant No. JCYJ20190809143601759); the National Natural Science Foundation of China under Grant 82261138629; Guangdong Basic and Applied Basic Research Foundation under Grant 2023A1515010688 and Shenzhen Municipal Science and Technology Innovation Council under Grant JCYJ20220531101412030.

## Conflict of interest

The authors declare that the research was conducted in the absence of any commercial or financial relationships that could be construed as a potential conflict of interest.

## Publisher’s note

All claims expressed in this article are solely those of the authors and do not necessarily represent those of their affiliated organizations, or those of the publisher, the editors and the reviewers. Any product that may be evaluated in this article, or claim that may be made by its manufacturer, is not guaranteed or endorsed by the publisher.
