# Supplementary material for: MIB-ANet: A novel multi-scale deep network for nasal endoscopy-based adenoid hypertrophy grading
Source: Front Med (Lausanne). 2023 Apr 14;10:1142261. doi: 10.3389/fmed.2023.1142261 (PMC10140414; doi:10.3389/fmed.2023.1142261)
Supplement: Supplementary file 1 [file Data_Sheet_1.pdf]

Supplementary A: The structure of AlexNet, VGG16, ResNet50, GoogleNet and the proposed ANet

In **Table 1**, we compare the structure of four classic classification CNNs, i.e., AlexNet, VGG16, ResNet50, GoogleNet and the proposed ANet. AlexNet, consists of five convolution layers and 3 fully-connected layers, and is the winner of ImageNet Challenge 2012. VGG achieved the best accuracy on ImageNet Challenge 2014 and GoogleNet won the championship of ImageNet Challenge 2014. ResNet, a residual learning framework, won the 1st place of the ILSVRC 2015 classification task. Different from these four models, the proposed ANet contains five convolution layers and atrous convolutions are used in the first 3 convolution layers. Compared to these four classic networks, less feature maps (32, 64, 128, 256 and 512) are extracted for each convolutional layer. Due to the above design, ANet contains fewer parameter (1.7 MB) than AlexNet (54.3 MB), VGG16 (128.0 MB), ResNet50 (22.4 MB) and GoogleNet (5.3 MB).

Table 1 The structure of AlexNet, VGG16, ResNet50, GoogleNet and the proposed ANet.

Structure	ANet	AlexNet	VGG16	ResNet50	GoogleNet
input 256×256 adenoid images					
Conv Block 1	Atrous Conv3×3, 32, rate 2, padding 2<br>max pool, stride 3	Conv11×11, 9<br>max pool, stride 2	Conv3×3, 64<br>Conv3×3, 64<br>max pool, stride 2	Conv7×7, 64<br>max pool, stride 2	Conv7×7, 64<br>max pool, stride 2
Conv Block 2	Atrous Conv3×3, 64, rate 2, padding 2<br>max pool, stride 3	Conv5×5, 256<br>max pool, stride 2	Conv3×3, 128<br>Conv3×3, 128<br>max pool, stride 2	(Conv1×1, 64<br>Conv3×3, 64<br>Conv1×1, 256)×3	Conv1×1, 64<br>Conv3×3, 192<br>max pool, stride 2
Conv Block 3	Atrous Conv3×3, 128, rate 2, padding 2<br>max pool, stride 3	Conv3×3, 384	Conv3×3, 256<br>Conv3×3, 256<br>Conv3×3, 256<br>max pool, stride 2	(Conv1×1, 128<br>Conv3×3, 128<br>Conv1×1, 512)×4	Inception Block, 256<br>Inception Block, 480<br>max pool, stride 2
Conv Block 4	Conv3×3, 256<br>max pool, stride 3	Conv3×3, 256	Conv3×3, 512<br>Conv3×3, 512<br>Conv3×3, 512<br>max pool, stride 2	(Conv1×1, 256<br>Conv3×3, 256<br>Conv1×1, 1024)×6	Inception Block, 512<br>Inception Block, 512<br>Inception Block, 528<br>Inception Block, 832

					max pool, stride 2
Conv Block 5	Conv3×3, 512<br>max pool, stride 3	Conv3×3, 256	Conv3×3, 512<br>Conv3×3, 512<br>Conv3×3, 512<br>max pool, stride 2	(Conv1×1, 512<br>Conv3×3, 512<br>Conv1×1, 2048)×3	Inception Block, 832<br>Inception Block, 1024
Prediction Layer	FC-512<br>FC-4<br>Softmax	average pool<br>FC-4096<br>FC-4096<br>FC-4<br>Softmax	average pool<br>FC-4096<br>FC-4096<br>FC-4<br>Softmax	average pool<br>FC-4<br>Softmax	average pool<br>FC-4<br>Softmax
Number Of Parameters (MB)	1.7+	54.3+	128.0+	22.4+	5.3+

Supplementary B: The framework of MIB-ANet and definition of evaluation metrics

Classification backbone - ANet: ANet used atrous convolutions and 3 × 3 maxpoolings to extract features of adenoid hypertrophy. The architecture of ANet contained 5 convolutional layers. Due to the large adenoid region in grade 3 and 4 adenoid images, it was reasonable to use a large receptive field at the shallow layers for better adenoid feature extraction. Therefore, we employed the atrous convolutions with rate 2 at the first, second and third layers. Maxpooling operation was used for reducing network parameters after the convolution operation. Different maxpooling operation with stride 2 and stride 3 are tested, and maxpooling with stride 3 showed the best performance. After convolution layers, the feature map was flattened to a 512 dimension feature vector, which was sent to 2 fully-connected layers for label prediction.

Modified Inception Block (MIB): In order to extract features of both low-level and high-level adenoid hypertrophy, we proposed a **multi-scale receptive field** block - MIB (Modified Inception Block) based on Inception V4-A Block for better feature extraction. We tested the performances of different version of Inception Block, Inception V4-A Block showed the best performance. Similar as Inception V4-A Block, MIB used convolution kernels of different sizes to extract and fuse multi-scale features. The difference was that MIB used an atrous convolution with kernel size of 3×3 and dilated rate of 4 to replace the stacked 3×3 convolution blocks of Inception Block, and used an atrous convolution with kernel size of 3×3 and dilated rate of 2 to replace the 5×5 convolution blocks of Inception Block. In addition, MIB didn't use BN (Batch Normalization) layer.

Loss Function: We utilized cross entropy loss to measure the divergence between predictions and ground truth, which was defined as:

$$cost(x) = -\frac{1}{N}\sum_{i} y_i log(p_i) . \#(1)$$

where N was the number of images in data set, y_i was the ground truth of image i, p_i was the probability of each grade predicted by network for image i.

Accuracy:

$$accuracy = \frac{TP + TN}{TP + FP + TN + FN} . \#(2)$$

where TP, FP, TN, and FN means True Positive, False Positive, True Negative and False Negative, respectively.

F1 score is a trade-off between precision and recall, which is defined as:

$$F1\ score = \frac{2 \times precision \times recall}{precision + recall} . \#(3)$$

$$precision = \frac{TP}{TP + FP} . \#(4)$$

$$recall = \frac{TP}{TP + FN} . \#(5)$$

Supplementary C: Details of classification performance evaluation (ablation study)

1. Ablation study of ANet

In order to justify the effectiveness of the operation used in ANet, we conduct the following ablation study on SYSU-SZU-EA Dataset.

Network depth: The number of convolution layers for feature extraction is a key for model performance. In this work, we train 3 versions of ANet for comparison, which consists of 3, 4 and 5 convolution layers, respectively. **Table 2** shows the classification performance. One can observe from the table that ANet with 5 convolution layers achieves the highest F1 score (0.67466) and accuracy (0.67871) among 3 versions of ANet with different depth.

Kernel size of max pool: Using larger kernel size of max pool can improve network performance for adenoid hypertrophy grading task. From **Table 2**, we can see that ANet with kernel size 3 achieves 6% higher F1 score and accuracy than ANet with kernel size 2.

Dilation rate: One can observe from **Table 2** that the network performance improves when the dilation rate increases from 1 to 2, but the network performance decreases when it increases from 2 to 4. It shows that properly increasing the dilation rate of convolution layer can improve the network performance, but further increasement will reduce the network performance.

Table 2 Ablation studies of ANet.

Network depth	Kernel size of max pool	Dilation rate	F1 score	Accuracy
3	2	1	0.60705	0.62149
4	2	1	0.66168	0.66667
5	2	1	0.67466	0.67871
5	3	1	0.74332	0.74900
5	3	2	**0.75302**	**0.75803**
5	3	4	0.73777	0.74096

2. Ablation study of MIB-ANet

We replace the layers of ANet with the proposed MIB. The performance of different versions of MIB-ANet with different number of MIBs is evaluated.

Number of MIBs: The number of MIBs for feature extraction is a key for model performance. In this work, we train five versions of MIB-ANet for comparison, which use 1, 2, 3, 4 and 5 MIBs to replace the convolutional block of ANet, respectively. **Table 3** shows the classification performances of MIB-ANet (the kth convolutional block of ANet is replaced with MIB). One can observe from the table that MIB-ANet2 achieves the highest F1 score (0.76251) and accuracy (0.76807) among five versions of MIB-ANet with different number of MIBs.

	MIB-ANet 1	MIB-ANet 2	MIB-ANet 3	MIB-ANet 4	MIB-ANet 5	F1 score	Accurac y
W/MIB						0.75302	0.75803

✓					0.74602	0.74900
✓	✓				**0.76251**	**0.76807**
✓	✓	✓			0.73253	0.73594
✓	✓	✓	✓		0.73305	0.72691
✓	✓	✓	✓	✓	0.71933	0.72490

Table 3 Ablation studies of MIB-ANet.

Supplementary D: Comparison of the performance of MIB-ANet, ANet and four classic CNNs

We adopted F1 score, accuracy and confusion matrix as the evaluation metrics of classification performance. In this study, we compared the performance of MIB-ANet, ANet and four classic CNNs, i.e., AlexNet, VGG16, ResNet50 and GoogleNet (**Table 4**). Details of ablation study for Classification Performance evaluation are described in **Supplementary C.**

Table 4 The Performance comparison of MIB-ANet, ANet and other classic CNNs.

Networks	F1 score	Accuracy
AlexNet	0.74871	0.75100
VGG16	0.69946	0.69478
ResNet50	0.68530	0.67671
GoogleNet	0.72890	0.73394
ANet	0.75302	0.75803
MIB-ANet	**0.76251**	**0.76807**

Among the four classic CNNs, the classification performance of AlexNet is the best, which proved that the design of ANet relieved the over-fitting problem. Take F1 score and accuracy as examples, AlexNet achieved 1.98% higher F1 score and 1.70% higher accuracy than ResNet50, VGG16 and GoogleNet. Compared to all baseline CNNs, ANet achieved the largest F1 score and accuracy, which are respectively 0.43% and 0.70% higher than the best baseline CNN - AlexNet. Compared to ANet, MIB-ANet achieved 0.94% higher F1 score and 1.00% higher accuracy than ANet.

Figure 1 shows the confusion matrices of different neural networks. In confusion matrix, the value on the diagonal represents the number of image correctly classified at all levels. For E.N.T doctors, the classification of grade 2 and 3 adenoid hypertrophy is very important, because when the patient reaches grade 3 adenoid hypertrophy and has some symptoms of oral breathing, surgical treatment of patients is recommended. From **Figure 1** we can see that for grade 2 and 3 adenoid images, ANet showed the best performance among the five networks, i.e., ANet, AlexNet, VGG16, ResNet50 and GoogleNet. In contrast, for grade 3 adenoid hypertrophy, MIB-ANet has comparable classification performance to ANet, while MIB-ANet in general achieved better classification performance than ANet, especially for grade 1, 2 and 4 adenoid hypertrophy.

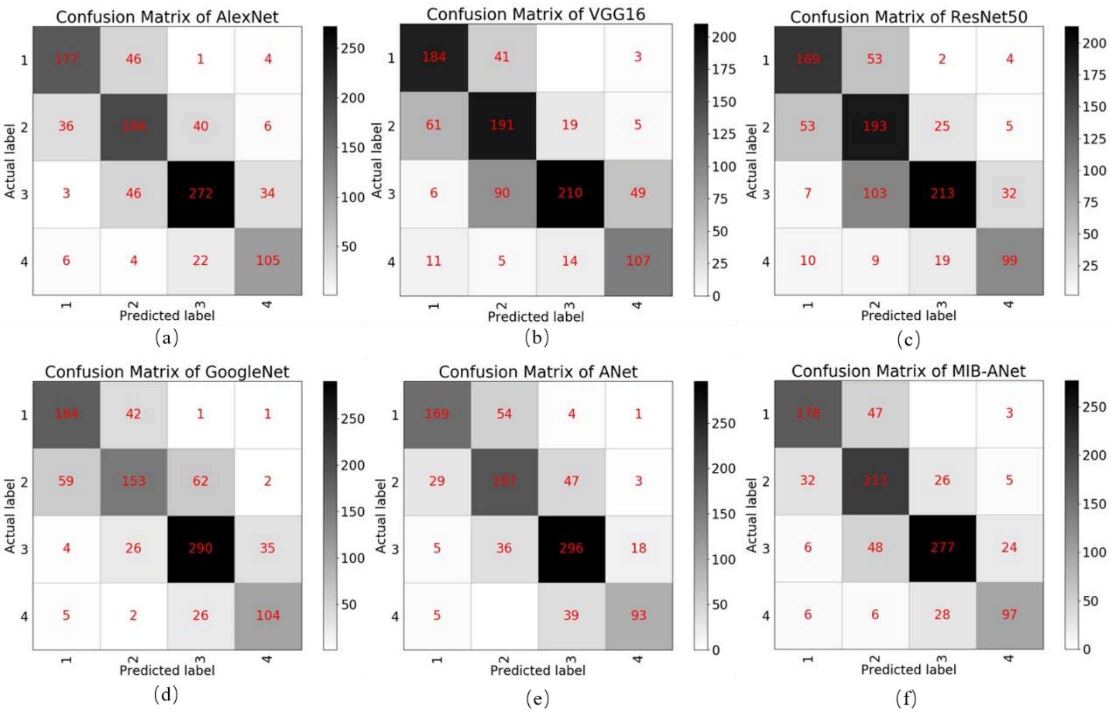

Figure 1 Confusion matrices of different deep networks. (a), (b), (c), (d) , (e) and (f) shows the confusion matrix of AlexNet, VGG16, ResNet50, GoogleNet, ANet and MIB-ANet, respectively.
